# An experimental characterization of workers’ behavior and accuracy in crowdsourced tasks

**DOI:** 10.1371/journal.pone.0252604

**Published:** 2021-06-16

**Authors:** Evgenia Christoforou, Antonio Fernández Anta, Angel Sánchez

**Affiliations:** 1 Transparency in Algorithms Group, CYENS - Centre of Excellence, Nicosia, Cyprus; 2 IMDEA Networks Institute, Leganés (Madrid), Spain; 3 Grupo Interdisciplinar de Sistemas Complejos (GISC), Departamento de Matemáticas, Universidad Carlos III de Madrid, Leganés (Madrid), Spain; 4 Institute UC3M-BS of Financial Big Data (IBiDat), Universidad Carlos III de Madrid, Getafe (Madrid), Spain; 5 Instituto de Biocomputación y Física de Sistemas Complejos (BIFI), Universidad de Zaragoza, Zaragoza, Spain; Universidad de Granada, SPAIN

## Abstract

Crowdsourcing systems are evolving into a powerful tool of choice to deal with repetitive or lengthy human-based tasks. Prominent among those is Amazon Mechanical Turk, in which Human Intelligence Tasks, are posted by requesters, and afterwards selected and executed by subscribed (human) workers in the platform. Many times these HITs serve for research purposes. In this context, a very important question is how reliable the results obtained through these platforms are, in view of the limited control a requester has on the workers’ actions. Various control techniques are currently proposed but they are not free from shortcomings, and their use must be accompanied by a deeper understanding of the workers’ behavior. In this work, we attempt to interpret the workers’ behavior and reliability level in the absence of control techniques. To do so, we perform a series of experiments with 600 distinct MTurk workers, specifically designed to elicit the worker’s level of dedication to a task, according to the task’s nature and difficulty. We show that the time required by a worker to carry out a task correlates with its difficulty, and also with the quality of the outcome. We find that there are different types of workers. While some of them are willing to invest a significant amount of time to arrive at the correct answer, at the same time we observe a significant fraction of workers that reply with a wrong answer. For the latter, the difficulty of the task and the very short time they took to reply suggest that they, intentionally, did not even attempt to solve the task.

## Introduction

Crowdsourcing systems are intended to bring together requesters, who have tasks they need to complete, with human workers, who are willing to perform them in exchange for a payment. Amazon Mechanical Turk (MTurk) [1] is the leading player in this market, and hence it will become the object of our focus hereafter. A requester announces a task in the MTurk platform in the form of a Human Intelligence Task (HIT), as they are called in MTurk, together with additional information on the task and the corresponding payment. Workers that like the task description accept to perform the HIT, and report back an answer when done. The requester then evaluates the collected responses, takes a decision on the task result and pays the workers.

Over the years, many studies tried to shed light on the traits of the crowd, analyzing the demographics of the participants [2–4], pointing out the sampling issues that might arise [5], elaborating on the motive of participation [6], and exploring how MTurk workers appraise their work and, consequently, the necessary economic incentives to establish a base quality of results [7, 8]. A number of works have shown that the observations from behavioral experiments carried out in a lab can be replicated using MTurk as a subject pool: Thus, Horton *et al*. [9] found that online experiments can be valid from both the internal and the external viewpoints, while Rand [10] provided further evidence with replications of more experiments and analysing self-reported demographics. On the other hand, there are also contradicting opinions [4, 5, 11, 12] regarding how reliable MTurk workers are, and whether they provide high quality and accurate data. Only in 2015, more than 1, 100 studies of this type were carried out with a total pool of about 30, 000 MTurk workers [13]. Also, Chandler and Paolacci showed that a substantial number of participants misrepresent some of their characteristics to meet elegibility criteria explicit in the studies done on MTurk [14]. Even so, subsequent results, such as the study of gender differences in altruism by Brañas-Garza *et al*. [15], have provided further examples of confirmation of laboratory results by using Mturk workers. In general, the available evidence shows both that high quality data can be collected from MTurk and that high quality data is by no means guaranteed [16].

These issues are not something affecting exclusively work done on MTurk. Indeed, recent events have shown that even in a proprietary polling platform (i.e., a special type of crowdsourcing platform) managed by the Organisation for Economic Co-operation and Development (OECD) [17], the reliability of the results depends upon the participants’ behavior. As it is mentioned in the official announcement [18] justifying the exclusion of a certain amount of data, it was noticed an “implausible student-response behaviour.” The reason behind the exclusion of the data collected in Spain in the reading category during PISA 2018 [19] is the unrealistic recorded time with respect to the task difficulty. This is a crucial point that is highlighted and used in this work as well to classify the workers’ behavior. Another context in which issues with fraudulent answers appear when obtaining online data is that of psychological clinical research, where it has been observed [20] that poor-quality responses can lead to several data-quality problems including spurious associations between measures.

In the above context, the research question we address in this paper is the characterization of online workers behavior as a function of the difficulty and type of the task in the absence of control techniques. It is clear that, due to the online nature of the system, a requester has little control on the workers choosing to perform her HIT, and has no detailed information on the crowd that undertook it [14]. In practise, requesters have a few tools they can use to augment their control and thus aspire for higher quality data. For example, it is possible to perform a pre-screening of the workers [21], consequently allowing or not their participation in the HIT. This practice has several shortcomings. On the one hand, it is hard to obtain an accurate knowledge on the workers’ skills, and thus valid workers can be excluded. On the other hand, it increases the completion time of the HIT (a specific amount of workers is usually necessary for each HIT). It has to be mentioned, however, that using the reputation tool provided by Amazon to select participants largely increase the quality of the answers [22, 23]. Another popular practice is to ask questions with a known (to the requester) answer (a.k.a, control questions), to decide on the trustworthiness of the data provided by that worker [3, 12, 24], to reinforce the good behavior [25, 26] of the worker, or to check how attentive the worker is [27]. Practically, in some HITs it can be hard to design such questions; and additionally, workers can become aware of these control questions and propagate the information to the community. In any case, one must be careful with how much of a worker’s valuable time these questions will consume, and how they will impact the payment [8].

A complementary approach to the implementation of control techniques is to analyze the performance of workers using different methods to identify potentially careless responses. Various approaches in the literature exist trying to evaluate how reliable crowdsourcing workers are, what affects their behavior, and how this reflects on the quality of the collected data [28]. Generally speaking, they consider the appropriateness of the economic incentives, their performance on specifically designed questions, or the validity of their answers in different types of HITs. In what follows we briefly present these three options and discuss how our experiment contributes to increasing our understanding of online workers’ behavior.

A first approach to deal with these possible problems is to think in terms of the economic incentives or payments to the workers. Indeed, the work of Suri et al. [29] shows that workers might cheat, reporting an incorrect answer, in an attempt to gain more economic benefits. Mason and Watts [30], on the other hand, saw that the “type” of the compensation scheme had an effect on the quality of the collected results. An alternative way to mitigate the problems we are discussing is to resort to quality checks based on the time workers take to complete specifically designed tasks or questions. In this respect, Downs et al. [27] considered a HIT with a screening question and presented correlations on the behavior of workers *providing wrong answers* and their occupation. They also recorded the time spent on two screening questions (an easy question and a difficult question), but it was not enough to be used as a tool for identifying *those careless or low-effort workers*. Kazai et al. [31], on the other hand, considered the completion time of the entire HIT, the worker’s accuracy, and the usefulness of the responses, in order to classify the workers into five categories. These were then associated with five personality traits, in an attempt to identify the most appropriate workers for a given job, and to find means to attract their participation. Further evidence of the effectiveness of response time to identify participants not working carefully enough was provided by Huang *et al*. [32], who identified it as one of the strongest indicators in this regard. Eickhoff et al. [33] proposed a solution facing the issue of problematic workers a priori, by designing tasks that are less attractive to them. Finally, a third option that has been proposed to ensure quality participation relies on analysing the quality of and factual correctness of their answers when possible. Thus, Difallah et al. [34] showed the limitations of existing techniques that are used to detect dishonest answers coming from either individual workers or groups of workers aiming at attaching the validity of a task result. Eickhoff et al. [35] reviewed how workers provide wrong answers in open and closed form questions. Through experimentation they tried to see the extent to which crowdsourcing workers attempt to cheat the system. They concluded that understanding worker behaviour better is a necessity. Gadiraju et al. [36] proposed a microtask classification. In a later work, Gadiraju et al. [37] focused on survey tasks and studied the behavior of workers, defining and identifying different types of workers.

Our contribution to advancing the knowledge in this field is related to the characterization of the workers behavior used the time they spent in the tasks and the correctness of their answer. Our aim in this work is not only to aid the requester to address the HIT to the most desirable workers, but also help the requester understand the behavioral patterns of the workers, and thus improve the quality of the received data a posteriori. To this end, in the present work we have chosen to give a fair compensation to workers, according to the work of Horton et al. [8]. This compensation is based on the time an honest and slow worker would take to compute the HIT, and therefore we can in principle exclude the lack of economic incentives as a relevant factor to understand the behavior of the participants in our experiment. With this caveat in place, we then build on the ideas of Downs et al. [27], by recording the time spend on each the question (sub-task) of our HIT. As we will see below, under certain conditions this time can be a good indicator for characterizing the worker’s behavior. In addition, in our experiment we will use both open and closed form questions, asking the workers to find some information, verify an information, or interpret and analyze an information. By doing so, our work adds on the work by Gadiraju et al. [37], and in fact through our HIT questions we explore the workers’ general behaviour in three out of the six categories of microtasks specified in [36].

## Materials and methods

### Ethics statement

Even though the data were analysed anonymously, the study has received approval from the ethics committee of IMDEA Network Institute. Participants selecting to complete one of our HITs on MTurk were consenting to participate in the study.

Our aim is to study the crowd of MTurk in an environment free from extra monetary incentives, instructions that might guide the workers’ behavior, a priori control techniques, and HITs that might be familiar to the workers. To this end, workers were asked to do one of three HITs or tasks, and for each task participants had to answer questions about four graphs of different levels of complexity. Participation was open to U.S. workers (December 2016 to March 2017) and by selecting one of the HITs they were consenting to participate in the study. As we never used any personal data from them, the Ethics committe of IMDEA Networks waived the need for further consent. All workers participating in the studies were rewarded and the demographics presented in S1 Appendix were collected in a voluntary base. Each HIT was completed by 100 workers and no worker participated more than once in the study, thus 300 distinct responses were received. Each HIT was posted subsequently once the previous HIT was completed (100 workers responded) in the following order: HIT Color, HIT Majority, HIT Count. All the data files are available from the Zenodo database (https://doi.org/10.5281/zenodo.3548689). No post-processing was done to identify “bots” as this was not permitted by the nature of our task, but our results referirng to some workers providing hasty wrong answers might also point to the use of “bots” by workers in the simplest of our HITs.

**Fig 1:**
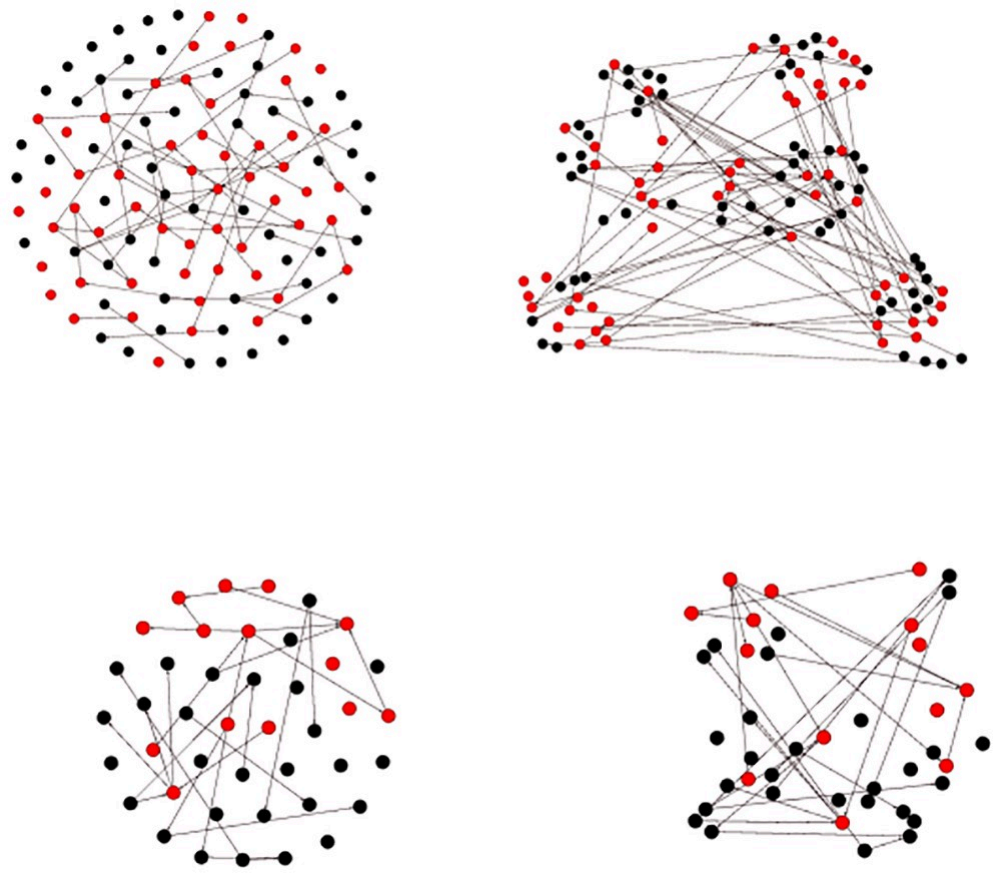
Graphs shown in the HITs subtask. Graphs G1 (top, left) and G2 (top, right) are different presentations of the same graph with 59 black nodes and 55 red nodes. Graphs G3 (bottom, left) and G4 (bottom, right) are different presentations of the same graph with 28 black nodes and 14 red nodes.

As already stated, each HIT consists of four sub-tasks, and in each sub-task questions about one of four network graphs presented in Fig 1 are asked. The nodes in each of the network graphs are either colored red or black. It is important to note that all network graphs were shown to every worker in a random order. The particularity of these graphs is that in pairs of two they have the same nodes with the same edges distributed in a different way. Network graphs G1 and G2 are the most complex ones, with 59 black nodes and 55 red nodes, while network graphs G3 and G4 are easier to analyze, with 28 black nodes and 14 red nodes.

The questions presented in the four subtasks (i.e., about each of our four graphs) in each task were the following:

- **HIT Color**: We ask the workers whether the red nodes or the black nodes are the majority in the graph.
- **HIT Majority**: We inform the worker that, according to the currently received answers, the majority of nodes in the graph are of a certain color. Then we ask the worker whether she agrees with this, and in case she does not agree we ask her to count the number of nodes of the other color.
- **HIT Count**: We ask the workers to let us know the total number of nodes in the graph.

The idea behind these HITs is to evaluate the workers’ behavior in different types of questions that are objective by nature but still require a worker’s cognition and engage the worker in finding some information, verify an information or interpret and analyze an information. We pose open and closed form questions in such a way that unique behavior characteristics of workers deviating from an honest behavior could be studied. In the HIT Color, we posed a closed form question with a binary answer, thus guessing behavior might arise. In the HIT Majority, the workers might simply choose a default solution without doing the effort of counting the nodes, that is, they are given an easy way out question. Finally, in the HIT Count, the workers must provide an accurate answer, which can expose spammers and low-quality workers (i.e., workers with limited skills or understanding of the task). We note that we have not included some of the standard HITs usually found on MTurk, such as finding information from the web, or collecting information from social networks. The reason for this is twofold. First, we would like to study the workers’ behavior in a controlled environment free from external influences, such as previous training or knowledge of the task. Second, we wanted to quantitatively assess the behavior of our workers’ which is hard to accomplish when dealing with subjective tasks. All in all, our setup allowed us to address the following questions:

- Do workers that provide wrong answers exist? Do they behave in that manner intentionally or unintentionally?
- Do such workers have a rationale for their behavior or they are pure spammers (provide random or uncorrelated answers with regards to the actual answer)?
- What are the possible criteria to separate workers intentionally providing wrong answers from those doing it unintentionally in the absence of control techniques?
- Can the requester benefit from this knowledge to improve the data quality, by matching responses to workers’ behaviors?

## Results

**Fig 2:**
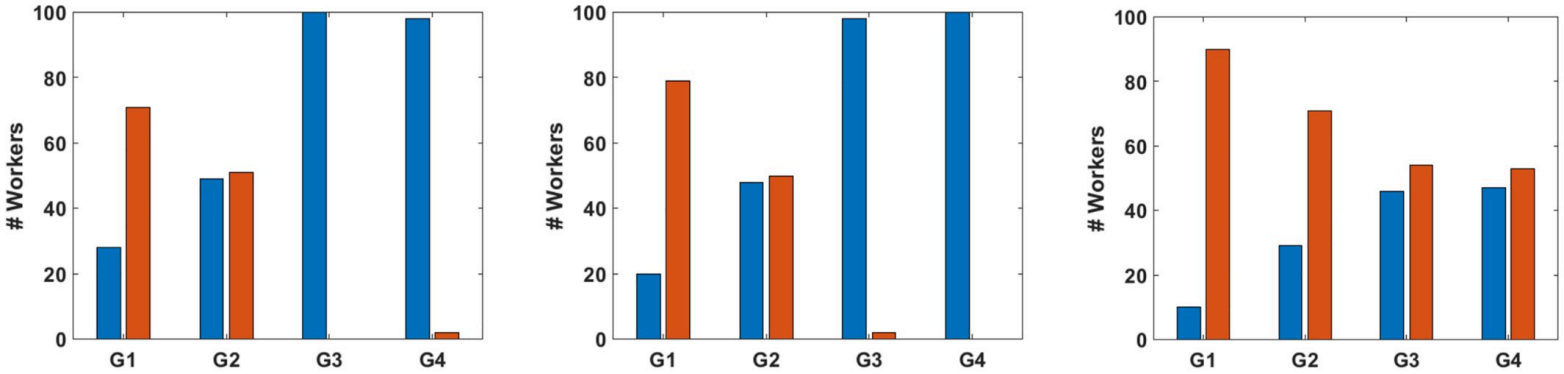
Percentage of workers’ correct and incorrect replies in all four sub-tasks for HIT Color (left), HIT Majority (middle) and HIT Count (right). In all three plots blue (left) bars represent correct replies and red (right) bars incorrect replies.

Fig 2 depicts the percentage of workers providing correct and incorrect responses in all four sub-tasks over all three HITs. As can be noticed, even for graphs G1 and G2, which are the most complex, for every HIT question there is a significant percentage of workers replying correctly. This is so even when we ask a question requiring accuracy in the answer. (E.g., even in HIT Count, in which the workers are asked to count the number of nodes, a 10% and 29% of the workers for graphs G1 and G2, respectively, reply with the correct answer.) Moreover, for simple questions (i.e., HIT Color and HIT Majority for graphs G3 and G4) almost all workers provide the correct answer, probably because the answer is quite obvious and also because there are only two possible answers. When the question is more difficult, like in HIT Count, the majority of the workers reply incorrectly, even in the two easy sub-tasks showing graphs G3 and G4.

It is also interesting to note that in HIT Color and HIT Majority the percentage of workers reporting a correct result in G1 is much lower than 50%, in comparison with G2 (different visualization of G1) where we almost received the same number of correct and incorrect responses. This is due to the fact that graph G1 has somehow a non-intended optical illusion. If the worker does not devote enough time to analyze the graph, she might easily believe that the majority color is red since red nodes are more concentrated in the middle of the graph and draw more attention. This is a first indication that some workers choose not to devote time to verify a seemingly obvious answer.

Let us now move to the question of whether there is any correlation between the time a worker uses to provide a reply and the correctness of the reply. To analyze this, we calculate the accuracy of each worker’s answer as the ratio of the worker’s correct sub-task responses over the number of sub-tasks. Our null hypothesis, for all three HITs posted, is that there is no linear correlation of the total response time of each worker with the worker’s accuracy. The correlation coefficients turn out to be 0.23 (HIT Color) with *p* = .01, 0.50 (HIT Majority) with *p* < .001, and 0.39 (HIT Count)with *p* < .001. As we can see, there is a significant correlation among a worker’s accuracy and her total response time.

However, an existing linear correlation between accuracy and response time does not give much information by itself. To gain further insight on this, we look at the Empirical Cumulative Distribution Function (ECDF) of the different accuracy groups in a HIT with respect to the workers’ total response time to the HIT. (Accuracy group *i* ∈ {0, …, 4} in a given HIT is the set of workers that returned exactly *i* correct responses over the four sub-task of the HIT.) We compute the ECDF according to the Kaplan-Meier estimate [38]. (This estimator is usually used for survival or failure times data, that is the time a certain element of a study remained active after a treatment, the time a machine part needs to fail, etc.) In our case we use this method to observe the time a worker needs to respond to the four sub-tasks.

**Fig 3:**
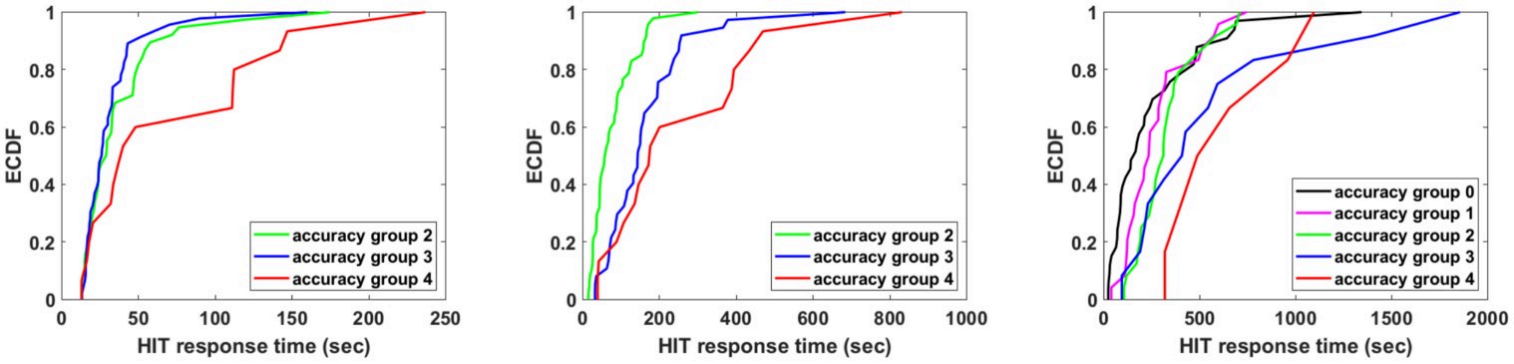
The ECDF of the total response time for HIT Color (left), HIT Majority (middle), and HIT Count (right). In each plot, from left to right, the curves are for workers with accuracy degree two up to degree four.

It is illuminating to discuss first the results for HIT Majority. As we see from Fig 2, accuracy group zero is not present while there are at most two workers in accuracy group one, which makes these two cases not interesting (and thus we do not include them in the plots). The results of Fig 3 show a large difference between accuracy group two and accuracy groups three and four. Combining these results with the ones of Fig 2, we observe that almost all workers replied correctly to the two easy tasks; while some of the workers where also able to give three correct responses, replying correctly to a difficult sub-task (in particular 37 workers), and 15 workers replied correctly to all four sub-tasks. Notice that for accuracy groups three and four, only 10% of the workers reply within the first minute, in comparison with accuracy group two, where around 60% of the workers reply in the first minute to all four sub-tasks of HIT Majority. This observation not only shows that workers’ accuracy is correlated with the response time but also indicates that a worker’s accuracy is correlated with the task difficulty.

Regarding the ECDF of the total response time for the HIT Color and each of the three accuracy groups (groups zero and one are empty, or have at most two elements), also shown in Fig 3, we observe that for accuracy groups two and three there is a large correlation among time and accuracy. More than 90% of the workers in these groups reply within 1 minute, while it takes the 90% of the workers in accuracy group four more than 2.5 minutes to reply. Notice that in all three accuracy groups a 25% of the workers replied within 30 seconds to all four sub-tasks. This observation suggests that a number of workers replied correctly simply because they guessed right, rather than actually counting the nodes. Comparing the plots for HIT Majority with HIT Color, we notice that the distinction between accuracy groups two and three is not very clear in HIT Color, pointing to a guessing behavior from the side of the workers.

Finally, we look at the ECDF of the total response time for HIT Count, shown in Fig 3. Notice that in this case, where a more difficult task is posed to the workers (one where they can not easily guess the correct answer), there is a positive correlation among accuracy and response time. Workers in accuracy group four needed at least 5 minutes to provide their four correct replies. We can also notice that there is a roughly constant separation in the plot among the five accuracy groups for values between 20% and 80%. This distance is likely to arise from the fact that the accuracy of a worker is linearly correlated with the response time. Notice that for values higher than 80% this correlation does not exist anymore. We believe that this is due to the fact that workers devote time in counting the nodes but they simply fail to provide the correct answer. On the other hand, for values below 10% it is very likely that workers in accuracy groups one, two, and three might have tried to guess the correct reply, instead of counting.

Let us now assess the correlation between the accuracy of the responses and the difficulty of the task, a relationship suggested by the three plots in Fig 3. We have hints of this relationship in all three tasks, each of which has 2 easy sub-tasks (those related to graphs G3 and G4) and 2 difficult sub-tasks (those about graphs G1 and G2). Further support for this relationship is provided by Table 1, which also shows that the accuracy is not affected much when a question regarding the nodes’ color is posed in two different ways (i.e., HIT Color and HIT Majority). Of course, this observation does not provide enough information, since the worker replying negatively to our question can still provide the wrong number of nodes in the majority. The difference among the three HITs is that, while HIT Color and HIT Majority ask a multiple choice question, HIT Count asks a question where the response is a positive integer, thus harder to guess. Fig 3 along with Fig 2 clearly show that, for HIT Color, accuracy depends on the difficulty of the sub-task. Sub-tasks with graphs G3 and G4 are easy to spot and thus all workers responded correctly. Conversely, for sub-tasks with graphs G1 and G2 it is more difficult for a worker to compute or guess the correct response, hence less workers reply correctly to them. This observation is also backed up by the response time as shown in Fig 3. Some of the workers in accuracy group three either guessed for graphs G1 and G2, or devoted time only to one of the two graphs, and thus the response time for group two and three is very similar.

**Table 1:** The number of workers belonging to each accuracy group in all three HITs.

	HIT Color	HIT Majority	HIT Count
Group zero	0	0	34
Group one	1	1	24
Group two	38	47	24
Group three	46	37	12
Group four	15	15	6

S14 (a) and S15(a) Figs in S1 Appendix shows the distribution of the answers of the workers that replied negatively to the requester’s question in HIT Majority in graphs G1 and G2 respectively. In the sub-task regarding graph G1 only 25% of the workers that replied negatively found the correct answer, while in the sub-task regarding G2, 37.5% of the workers that replied negatively found the correct answer. If we compare these results with the histograms for the HIT Count, we can see that, in the case of graph G1 (c.f., S16 (a) Fig in S1 Appendix), only 10% of the workers replied correctly, while in the case of graph G2 (c.f., S17 (a) Fig in S1 Appendix), 29% of the workers replied correctly. In the above mentioned cases, we could say that the task difficulty has to do with the number of nodes needed to be counted, that is black nodes v. all the nodes. In order to test in this case if accuracy depends on this kind of task difficulty, we have our null hypothesis assume that it does not and we compute a two proportion z-test for HIT Majority and HIT Count for graph G1 and G2 separately. In the case of G1 the *z* = 1.85 with *p* = .06 and for G2, *z* = 1.04 with *p* = .29. Thus, when it comes to counting based on color v. simply counting this is not significant and accuracy doesn’t depend on this kind of “visual” task difficulty.

As we have noticed before in Fig 3, 30% of the workers in HIT Color and in all three accuracy groups examined answered all four sub-tasks within 30 seconds, while 80% of the workers that had full accuracy, i.e., correct response ratio one, replied within 2 minutes. This suggests guessing behavior from the fast answering workers, with some of them being able to guess correctly. In the case of HIT Count, even the fastest workers that had full accuracy needed almost 5 mins to respond to the requester, as we see in Fig 3. Hence, we can conclude that many of the workers that have full accuracy in HIT Color did not count the nodes, but rather devoted time in guessing the correct answer.

Finally, the values summarized in Table 2 allow us to identify to what extent workers may be responding with a guess instead of carrying out the requested task. Table 2 shows the correlation coefficient values between the worker’s response time in each graph and its correct response ratio (in the four sub-tasks). It is immediately obvious for graphs G3 and G4, which are the easy graphs, and HITs Color and Majority, which are HITs that allow guessing, the correlation is very low. Thus, it is clear that highly accurate or not, workers are responding in roughly the same time, which is very low as we have already observed. This is a clear indication of guessing the answer in these easy graphs. Another thing that we can notice from Table 2 is the high correlation in the difficult tasks G1 and G2 in HITs Majority and Count. Workers with high accuracy invest also a lot of time in these graphs, which makes us conclude that correct guessing behavior in these graphs is smaller. Table 3 supports the argumentation that workers are prone to guess, since for all graphs there is a negative correlation between the distance from the correct answer in HIT Count and the response time.

**Table 2:** The correlation coefficient of the workers’ correct response ratio (in the four sub-tasks) with the response time in each graph. Columns represent the correlation coefficient for each of the graphs and rows represent the HIT task.

	G1	G2	G3	G4
HIT Color	0.29	0.19	0.05	-0.001
HIT Majority	0.49	0.38	0.13	0.23
HIT Count	0.41	0.30	0.40	0.10

**Table 3:** The correlation coefficient of the workers’ correct response ratio (in the four sub-tasks) with the absolute distance to the correct value for HIT count. Columns represent the correlation coefficient for each of the graph and rows represent the HIT task.

	G1	G2	G3	G4
HIT Count	-0.16	-0.34	-0.26	-0.21

### Criteria for categorizing a worker’s behavior

To analyze the workers’ behavior we have used as criteria the number of correct responses a worker has provided in a HIT, the time it took her to reply to each sub-task of the HIT, the difficulty of the HIT, and the type of microtask/question asked. We have noted different worker behaviors based on these criteria. Some workers appear to exhibit a rational behavior and gave a wrong answer to save time, consequently earning more money (by moving on to complete other HITs), while other workers seemed to have more of a spamming behavior. We also a number of workers unintenionlly providing wrong answers, since they invested a large amount of time to complete all the sub-tasks and have failed in the most difficult one. Finally, we also observed that some workers invested a large amount of time to provide correct answers. A clustering analysis based on the workers’ behavior (c.f., S1 Appendix) shows also that workers can be grouped in three main categories, workers intentionally providing wrong answers, workers doing so unintentionally, and workers that make an effort to give the correct answer, thus supporting and complementing our observations above.

**Fig 4:**
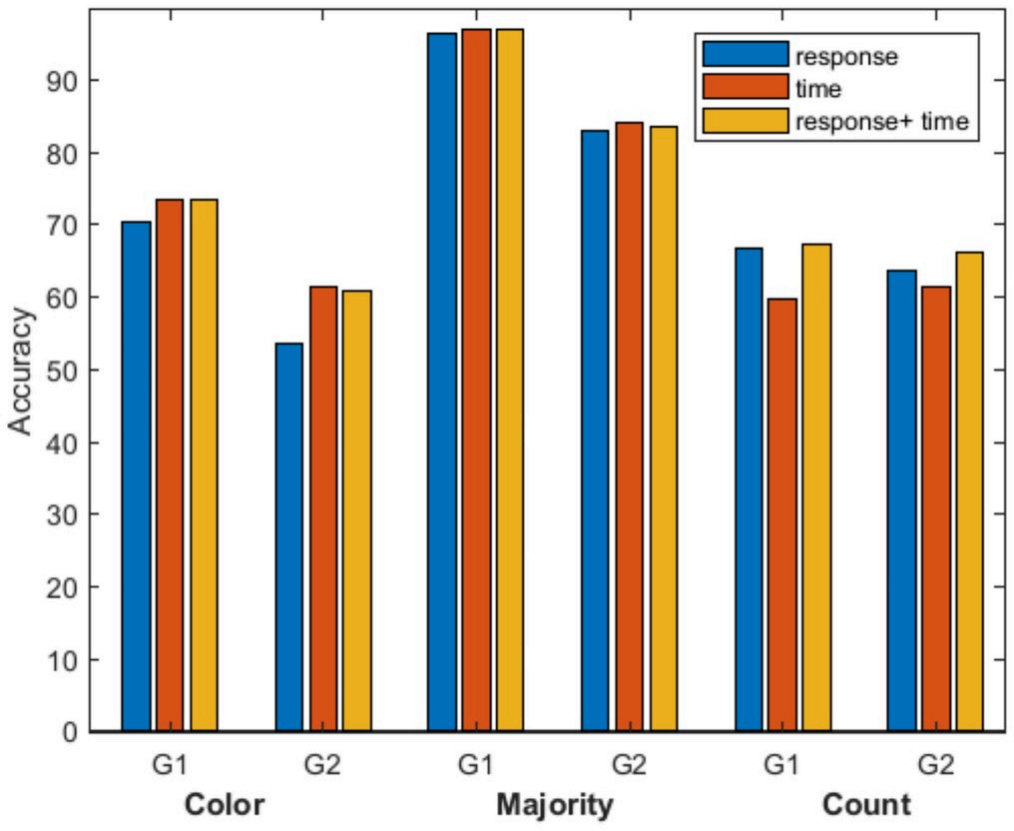
Prediction accuracy of the best classification Learner. Predicting the workers responses for graphs G1 and G2 for all three HIT, using responses, time and response plus time as predictors.

Now we ask a different question, whether we could use the same criteria we used to judge the workers’ behavior to predict whether the worker would reply correctly or incorrectly. We tried to predict the response of the worker for the two difficult graphs (G1 and G2) when the only criteria is (a) the responses of the worker for the other two graphs in the three sub-tasks, (b) the time the worker invested for the other two graphs in the three sub-tasks, (c) the response given and the time the worker invested for the other two graphs in the three sub-tasks. Results are shown in Fig 4 and reflect the best accuracy obtained after running all the classification learners provided by the Matlab Classification Learner [39] package for the predictors and criteria mentioned above. Our results, summarized in Fig 4, indicate that responses in the other graphs and response time allow to predict the worker’s answer better than random, with accuracies between 50% and 90%, most of the cases being at the level of 70%). Particularly in difficult HITs, like in HIT Count, considering only the time factor is not enough and the responses of the workers must also be taken into account. This suggests that in difficult tasks with open ended questions time becomes a more subjective factor.

### Replication

Seven months (October-November 2017) after finishing this first study, we run a replication study with exactly the same HITs and number of participants. The sets of workers participating in each of the studies were disjoint (for a total of 600 distinct workers between the first study and its replication), within the guarantees provided by MTurk regarding unique workers’ identifications. The results of the replication turned out to be strikingly similar to those reported above (c.f., S1 Appendix), which is a strong indication of the robustness of our results. The aggregated results collected from both studies are also presented in S1 Appendix and as can be seen they fully support our findings above.

## Discussion

We have evaluated the behavior of MTurk workers for a set of tasks that require the workers’ attention, but are neither self-reporting nor pose factual questions that can be searched on the Internet. We posed cognitive tasks with different levels of difficulty to the workers and asked closed form questions, semi-closed form questions, and open form questions. In each HIT one category of the above questions was asked regarding two different representation of two graphs with different levels of difficulty (i.e., the sub-tasks of the HIT). We measured difficulty in terms of the nodes in the graph and consequently, the time needed for a human to process them.

We have observed that, regardless of the difficulty, some workers answer correctly to the sub-tasks, even if that implies investing more time than what would seem reasonable. On the other hand, also regardless of the difficulty, some workers will reply incorrectly. The workers’ accuracy is correlated with the difficulty of the sub-task and with the time they invest in solving it. It is interesting to note that for a sub-task with a binary answer we obtained 70% of incorrect answers (when a random guess would have given 50% on average). This finding, along with other facts such as the low accuracy and short response time of certain workers, allows us to conjecture that a number of workers is guessing the correct answer instead of accurately calculating it. We have also noticed that monitoring a worker’s response time can be a valuable predictor, when using a machine learning approach to verify a worker’s answer. Importantly, the fact that the study was replicated after seven months and that the results were practically the same with different subjects makes us confident that these conclusions are robust.

The implications of our work touch upon different issues arising in dealing with the possibility of receiving wrong answers when resorting to online job markets such as MTurk. Thus, our results are aligned with those of [27], our primary source of inspiration, but in our case we use time response to detect wrong answers in the task themselves, not as a screening tool. In this respect, our findings are in agreement with those of Huang *et al*. [32], and give further support to the use of time response as a tool to single out those workers who behave carelessly or do not exert the necessary effort. To this we add the idea of using the responses in other tasks to predict the accuracy of the one under consideration. The fact that this yields good results is not unrelated to the use of reputation to select the best workers for a task [22]. Interestingly, our setup has allowed to tentatively identify workers intentionally providing wrong answers, i.e., we have been able to probe into motivation and not only detect who is producing low quality work.

Clearly, our work has limitations as all experimental research does. To begin with, we have carried out our experiment on MTurk, and we have succesfully replicated on the same platform, but we do not know whether our intuitions about workers intentionally providing wrong answers will also be of use in other types of online platforms, let alone in other contexts. It should also be mentioned that even if our findings are suggestive of intention, they are by no means a rigorous proof, and therefore they should be taken as a motivation for further work along these lines rather than as a definitive answer. In this regard, it would be interesting to study how our procedures perform when combined with control techniques, in order to see whether or not controls affect differently the behavior of those workers identified as giving wrong answers intentionally as compared with others just making unintentional mistakes.

Our results can be useful for prospective users of crowdsourcing platforms. At a minimum, our findings hint that the design of the HITs is crucial towards optimizing the quality of the responses, and that it may be needed to break HITs down to simpler sub-tasks that allow to produce correct answers with less effort. This result aligns with suggestions along similar lines in [40]. Appropriate mechanisms to pay the workers should also be considered as a further source of quality improvement [26]. A third aspect that can be of interest for real life crowdsourcing application is the possibility of improving the quality of the collected results through post-processing. This may be even more relevant when lack of resources to pay skilled workers or a very short time frame for getting answers makes it necessary to accept answers from any possible worker. Finally, a particular context where our findings are relevant is that of behavioral or psychological experimental studies carried out with MTurk workers. The possibility that some participating subjects might give incorrect answers should be kept in mind when designing the experiment and the subsequent data analysis.

## Supporting information

S1 Appendix Supplementary information for this study. (PDF)
