# Supplementary material for: An experimental characterization of workers’ behavior and accuracy in crowdsourced tasks
Source: PLoS One. 2021 Jun 16;16(6):e0252604. doi: 10.1371/journal.pone.0252604 (PMC8208528; doi:10.1371/journal.pone.0252604)
Supplement: S1 Appendix — (PDF) [file pone.0252604.s001.pdf]

A sizable fraction of workers provides hasty, wrong answers in crowd-sourced tasks
Supplementary Material

Evgenia Christoforou, Antonio Fernández Anta, Angel Sánchez

November 1, 2020

This document provides supporting information on the design of the Human Intelligence Tasks (HITs) posted on Amazon Mechanical Turk (AMT). Also, information on the replicate study on AMT is presented with the supporting results and an aggregated report of the demographics of all 600 worker is shown. Furthermore, a complimentary analysis on the reported results, following a clustering approach, is presented.

Experimental Set-Up for both studies on AMT

Methods

HIT Color, HIT Majority and HIT Count were posted on AMT for each study (original and replica) showing the same four network graphs G1-G4, as shown in Fig. 1, in a random order. Below we present a comprehensive report of the values assigned to the HIT parameters, such as the task description, promised reward, number of assignments, allocated time, etc. Besides these parameter values which we are set before placing the HIT online, we have a number of internal task parameters. In particular, we record the worker's response to each of the four subtasks together with the time it took the worker to respond. Moreover, we record the workers responses on a few demographic questions: age, gender, education and occupation.

Notice that the payment in each HIT varies proportionally to its difficulty, in an effort to cancel out the effect that payments might have on motivating the workers honesty (or equivalently dishonesty). The three HITs designed are presented below in order of ascending task difficulty.

Color HIT Design

The initial preview of the HIT implementing the color variation is presented in Figure 2, while the HIT parameters are the following:

Title: Looking at network nodes;
Description: Find out whether the majority of nodes has the color black or red in a graph;
Keywords: graph, network, majority;
Rewards per assignment: \$0.10;
Number of assignments per HIT:100;
Time allotted per assignment: 1h;

HIT expires in: 7 days (with possibility of extending the initial expiration time);
Auto-approve and pay Workers in: 7 days;
Require that Workers be Masters to do your HITs: No;
Workers must: be from USA;
Workers must: not have participated in our experiments in the past.

If the workers accept to compute the task based on the HIT preview (see Figure 2) that gives them the instructions and an example of the task, then the HIT is presented to them, and the four graphs are shown to them in a random order. No default value, red or black is preselected for the workers. Notice that is not obvious which is the majority color for graph G1 while for G2 graph it is more obvious and for graphs G3 and G4 the majority color in the nodes is obvious.

Note that we do not provide any explanation to the workers as to the methodology we will use to reward them (based on the validity of all 4 replies or proportionally). Workers are only aware that completing correctly a task, according to the requesters standards, will be worth $0.10. We use such an approach for the following reasons: (1) To simulate the standard "vague" approach taken by the majority of the requesters. (2) To avoid workers strategically replying to questions if we were to reward proportionally for each correct answer to a subtask. (3) In case we were to reward only if a worker replied correctly to all questions, we would have actually given additional incentives to the workers to respond honestly, while our intention is to study their behavior.

Majority HIT Design

The initial preview of the HIT implementing the majority variation is presented in Figure 3, while the HIT parameters are the following:
Title: Looking at network nodes;
Description: Let us know your opinion on the majority color of the graphs shown;
Keywords: graph, network, majority;
Rewards per assignment: $0.15;
Number of assignments per HIT:100;
Time allotted per assignment: 1h;
HIT expires in: 7 days (with possibility of extending the initial expiration time);
Auto-approve and pay Workers in: 7 days;
Require that Workers be Masters to do your HITs: No;
Workers must: be from USA;
Workers must: not have participated in our experiments in the past.

In this HIT we inform the worker that the majority of the received answers claims that nodes with a particular color form a majority in the graph. We ask the worker to let us know whether she agrees with the majority. If the worker disagrees, she must compute the number of nodes in the majority. In particular, in the difficult subtasks showing G1 and G2, the question claims that the majority has reported red as the majority color in the graph. While, in the easy subtasks showing graphs G3 and G4, the question claims the truth that the nodes in majority are of color black. Aside from discussion in the previous HIT about the difficulty of identifying the majority without counting, now counting the number of nodes with different colors can be confusing in the case of graphs G1 and G2. Again, we do not provide any explanation to the workers as to the methodology we will use to reward them (based on the validity of all 4 replies or proportionally) for the reasons mentioned before. Workers are only aware that completing correctly a task, according to the requesters standards, will receive $0.15.

Count HIT Design

The initial preview of the HIT implementing the count variation is presented in Figure 4, while the HIT parameters are the following:

Title: Looking at network nodes;

Description: Let us know the number of nodes in a graph;

Keywords: graph, network, count;

Rewards per assignment: $0.20;

Number of assignments per HIT:100;

Time allotted per assignment: 1h;

HIT expires in: 7 days (with possibility of extending the initial expiration time);

Auto-approve and pay Workers in: 7 days;

Require that Workers be Masters to do your HITs: No;

Workers must: be from USA;

Workers must: not have participated in our experiments in the past.

Again, the workers that accept to compute the task based on the HIT preview (see Figure 4) are presented with the four graphs in a random order and are asked to count the number of nodes in the graph. The graphs G1 and G2 are obviously more difficult in comparison to G3 and G4. No further explanations are provided to the workers as to the methodology used to reward them besides the fact that if the reported task result is marked correct they will receive $0.20.

Demographics

After asking each worker in both studies to respond to four question on graphs G1-G4 we asked them to voluntary participate to a small demographics questionnaire. We have mentioned to the workers that replying to these demographics questions was not obligatory. As we have seen 47.6% of the worker population reported they are women, while 51.3% reported they are men and the rest of the workers choose not to reply. Figure 18 shows that the majority of workers are among 20-40 years old. In Figure 19 we can observe that more than half of the workers have received a higher education while in Figure 20 we see that a bit more than half of the workers don't have a fixed occupation.

Replication study: separate and aggregate results

This section presents the results collected in the replication study, Study 2, and compares them with the results of the original study, Study 1 and the aggregated results. Fig. 8 presents the number of workers' correct and incorrect replies in all four subtasks for HIT color. Fig. 9 presents the number of workers' correct and incorrect replies in all four subtasks for HIT majority. Fig. 10 presents the number of workers' correct and incorrect replies in all four subtasks for HIT count. Table 1 presents the correlation coefficient of the workers' response time with the accuracy of the workers (ratio of worker's correct subtask responses over all HITs and studies. Table 2 presents the number of workers belonging to each accuracy group in all three HITs, for Study 1. Table 3 presents the number of workers belonging to each accuracy group in all three HITs, for Study 2. Table 4 presents the correlation coefficient of the worker's correct response ratio (in the four subtasks) with the response time in each graph, for Study 1. Table 5 presents the correlation coefficient of the worker's correct response ratio (in the four subtasks) with the response time in each graph, for

Study 2. Table 6 presents the correlation coefficient of the worker's correct response ratio (in the four subtasks) with the response time in each graph, for the aggregate of the two studies. Table 7 presents the correlation coefficient of the worker's correct response ratio (in the four subtasks) with the absolute distance to the correct value for HIT count. Fig. 11 presents the ECDF of the total response time for HIT majority. Fig. 12 presents the ECDF of the total response time for HIT color for the three accuracy groups. Fig. 13 presents the ECDF of the total response time for HIT count for the five accuracy groups. Fig. 14 presents the histogram of the density of reported number of black nodes in graph G1 for HIT majority. Fig. 15 presents the histogram of the density of reported number of black nodes in graph G2 for HIT majority. Fig. 16 presents the histogram of the density of reported number of nodes in graph G1 for HIT count. Fig. 17 presents the histogram of the density of reported number of nodes in graph G2 for HIT count.

Categorizing workers' behavior

In order to classify the individual workers based on their behavior, we have used a clustering algorithm for each HIT. Each worker in a HIT is assigned a vector with the response time for each subtask in the HIT and whether the answer provided for the subtask was correct. The application of this algorithm resulted in aggregating the workers of a HIT into three clusters.

We have run a k-mean clustering algorithm [1] for each HIT with a vector of the workers response time and four parameters specifying whether the worker replied correctly or not to the subtask. The k-mean clustering algorithm we used uses the squared Euclidean distance measure and the cluster k-means++ algorithm for cluster center initialization as it is implemented in MATLAB [1]. For each HIT vector we have run the k-mean algorithm evaluating the number of centroids. The way we choose the optimal centroid value was through visualization of the created clusters by k-mean. We visualized the data by plotting the scatter plot of the total response time against the number of correct responses in each task. We have found that while 2 centroids were giving two almost distinct clusters it was not enough to provide us with any useful information. On the other hand, 4 and 5 centroids were too many and there was no clear meaning in the clusters. Thus, we concluded that 3 centroids are enough to provide us with sufficient information, without having clusters interfering too much with each other.

Figure 21 presents the response times and accuracy group of all the workers, identified by the Cluster to which they belong for the three HITs and the four subtasks. In particular, Figure 21.(a) presents this for the HIT color. This figure shows that Cluster 1 contains workers that invest a significant amount of time in answering the subtasks, and also answer correctly to most of them. The time invested in each subtask by the workers in this Cluster is correlated with the subtask difficulty, which seems to indicate that they perform the subtask indeed, and that their mistakes are accidental. Moreover, the time they invest is rather high given the subtask payments, which seems to indicate an altruistic behavior.

On the other hand, Cluster 3 contains workers that invest very small time in each subtask and, not surprisingly, have a low number of correct answers. Moreover, the amount of time they invest in a subtask does not seem to be influenced by the difficulty of the subtask, which leads to believe that they do not perform the subtask requested, and their answers are guesses. In fact, there are few workers in this cluster that answer correctly all subtasks. All this seems to indicate a greedy rational behavior for the workers in this cluster.

Finally, Cluster 2 contains workers that invest a time proportional to the difficulty of the subtask,

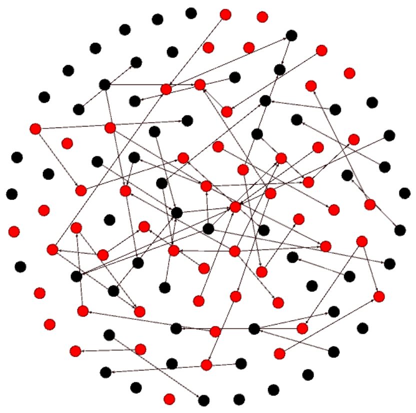

(a1) Graph G1

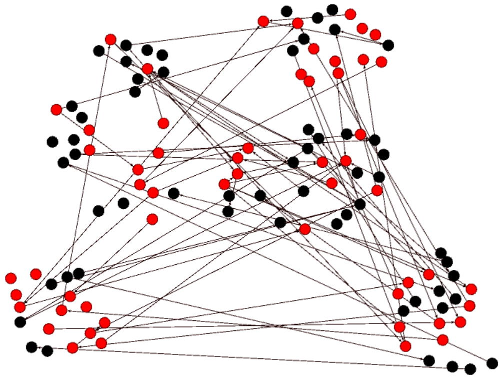

(a2) Graph G2

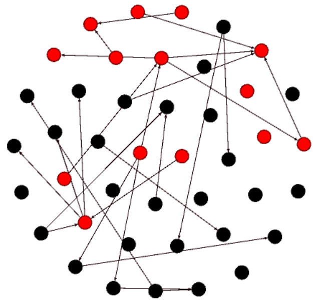

(b1) Graph G3

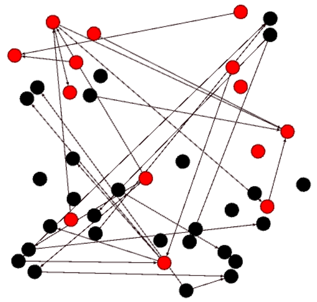

(b2) Graph G4

Figure 1: **Graphs shown in the HITs subtasks.** Graphs G1 and G2 are different presentations of the same graph with 59 black nodes and 55 red nodes. Graphs G3 and G4 are different presentations of the same graph with 28 black nodes and 14 red nodes.

but up to a limit. They seem to have a threshold on the time they will devote to each subtask, and hence they fail more often than workers in Cluster 1, but less than workers in Cluster 3. This seems to indicate a rational behavior but less greedy than in the case of workers of Cluster 3.

Figures 21.(b) and 21.(c) present similar results for the other two HITs, namely Majority and Count. The results show similar patterns as those identified in Figure 21.(a). It may be worth noting that the response times and the number of wrong answers is higher in the HIT count shown in Figure 21.(c). This figure shows that workers in Cluster 1 invest more time (up to more than

10 minutes) and get better answers than the rest (all workers except one get at least 3 correct answers), while workers in Cluster 3 invest less time and get worse answers (most of them get less than 3 correct answers). Workers in Cluster 2 are balanced among the different accuracy groups.

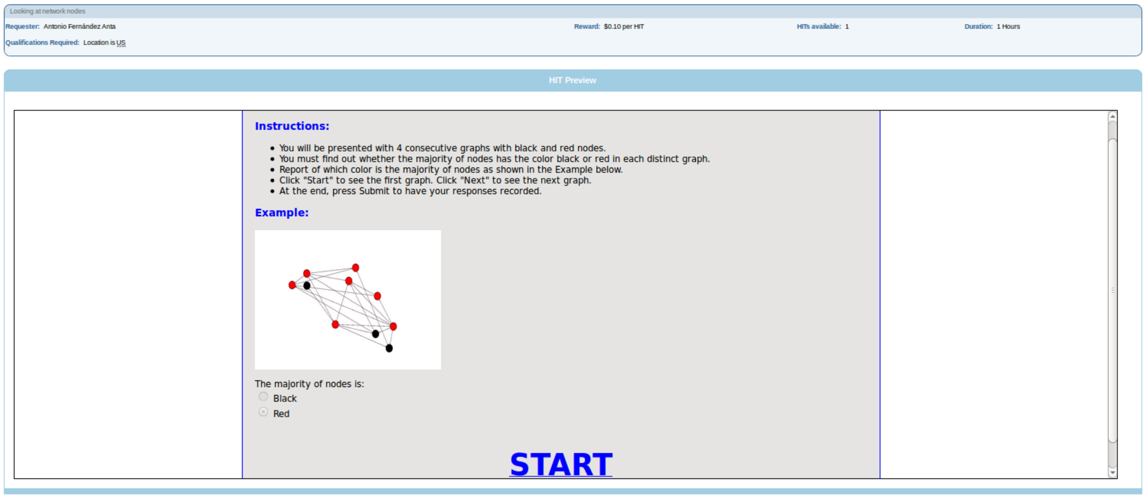

Figure 2: **HIT preview, for the task variation color, as seen by the worker.**

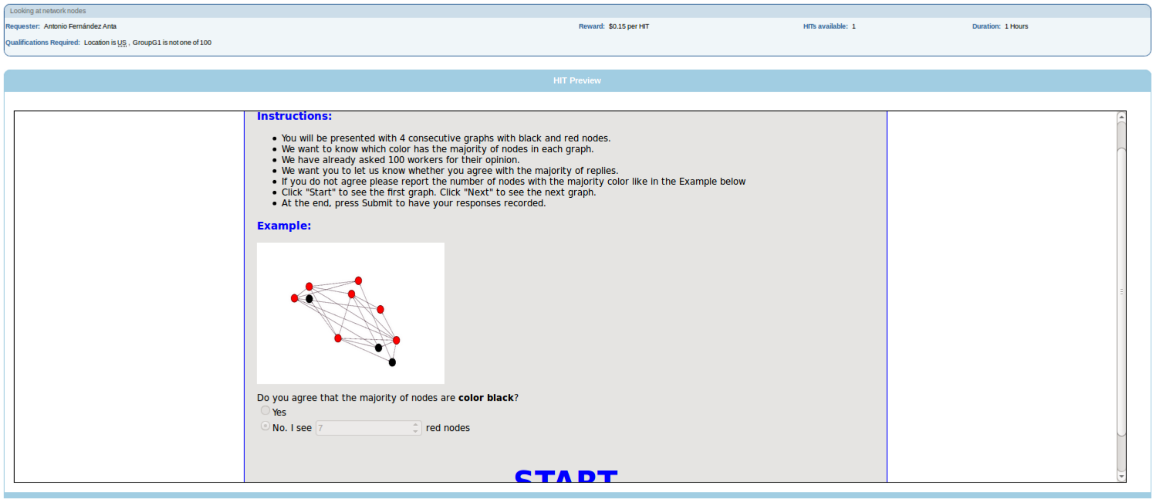

Figure 3: **HIT preview, for the task variation majority, as seen by the worker.**

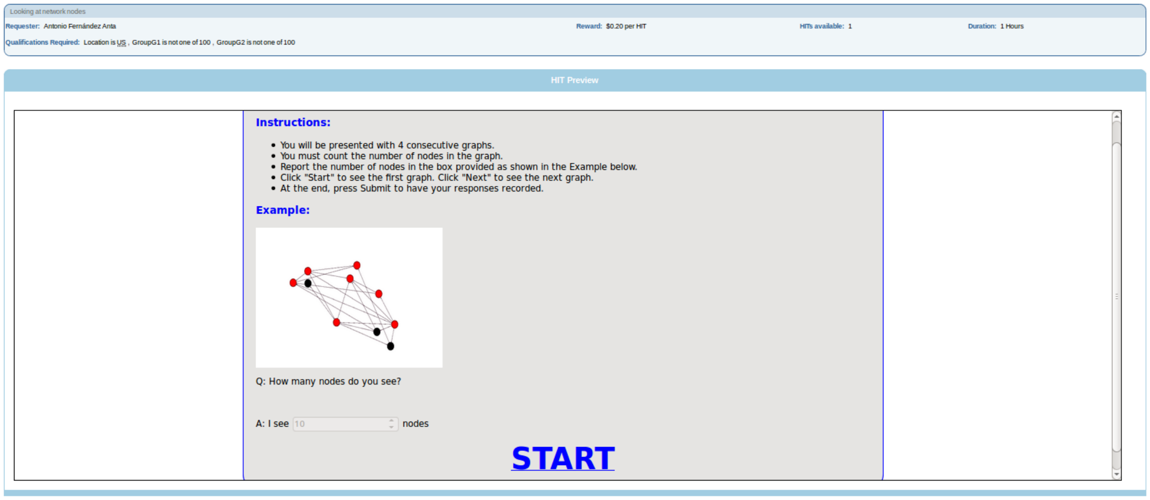

Figure 4: **HIT preview, for the task variation count, as seen by the worker.**

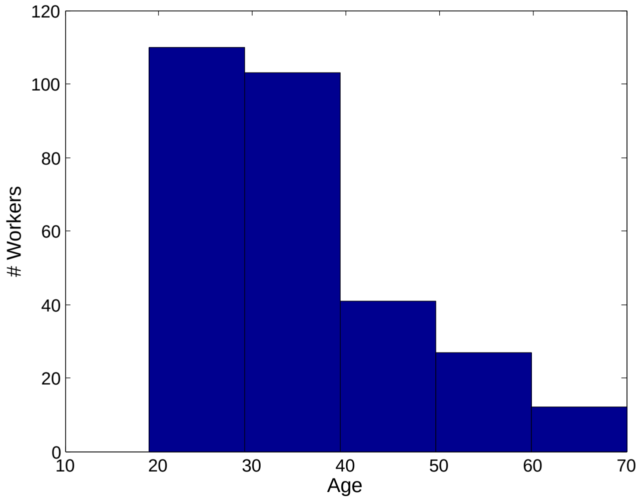

Figure 5: **The reported age of participating workers in all three HITs**

References

[1] David Arthur and Sergei Vassilvitskii. k-means++: The advantages of careful seeding. In *Proceedings of the eighteenth annual ACM-SIAM symposium on Discrete algorithms*, pages 1027–1035. Society for Industrial and Applied Mathematics, 2007.

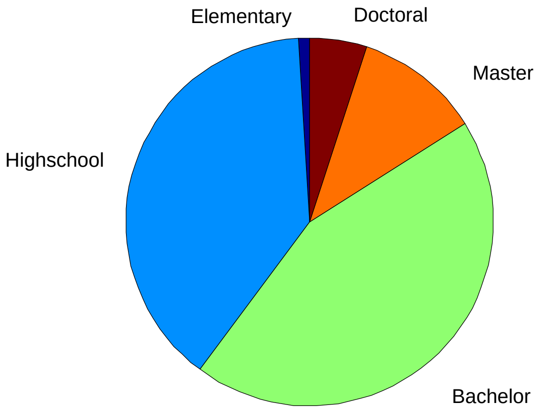

Figure 6: **The reported education of participating workers in all three HITs**

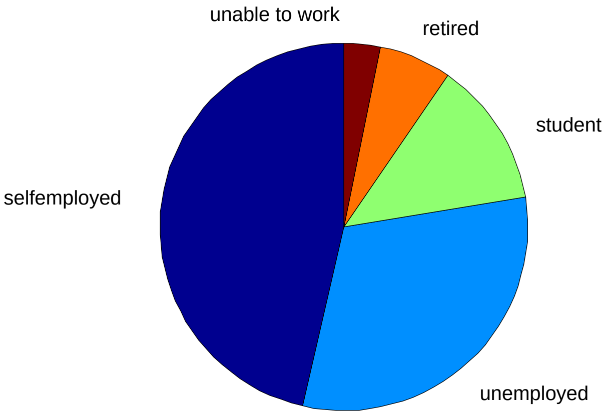

Figure 7: **The reported occupation of participating workers in all three HITs**

Table 1: **Correlation coefficient of the workers' response time with the accuracy of the workers (ratio of worker's correct subtask responses over all subtasks in the HIT.** Columns represent the three HIT tasks published on AMT.

Round	Color	Majority	Count
Study 1	0.2339	0.5024	0.3811
Study 2	0.4616	0.4328	0.4093
Aggregate	0.3568	0.4566	4003

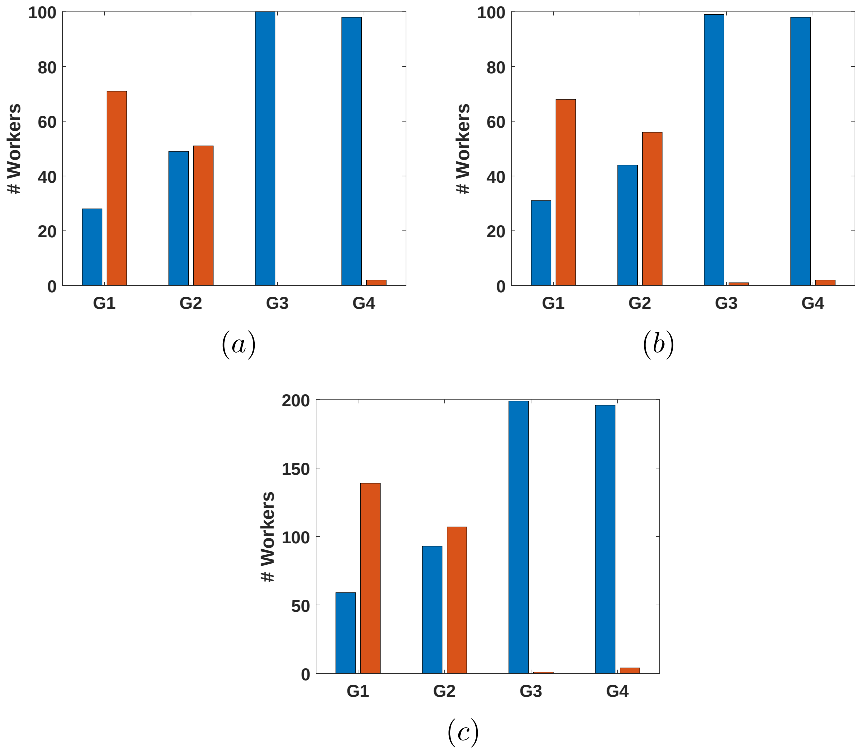

Figure 8: **Number of workers' correct and incorrect replies in all four subtasks for HIT color. (a) Results collected from Study 1, (b) results collected from Study 2, (c) aggregated results collected** We present with the blue color (left) bar the correct replies and with the red color (right) bar the incorrect replies.

Table 2: **The number of workers belonging to each accuracy group in all three HITs, for Study 1.**

	Color	Majority	Count
Group zero	0	0	34
Group one	1	1	24
Group two	38	47	24
Group three	46	37	12
Group four	15	15	6

Table 3: **The number of workers belonging to each accuracy group in all three HITs, for Study 2.**

	Color	Majority	Count
Group zero	0	1	38
Group one	3	1	31
Group two	36	54	12
Group three	47	29	10
Group four	14	15	8

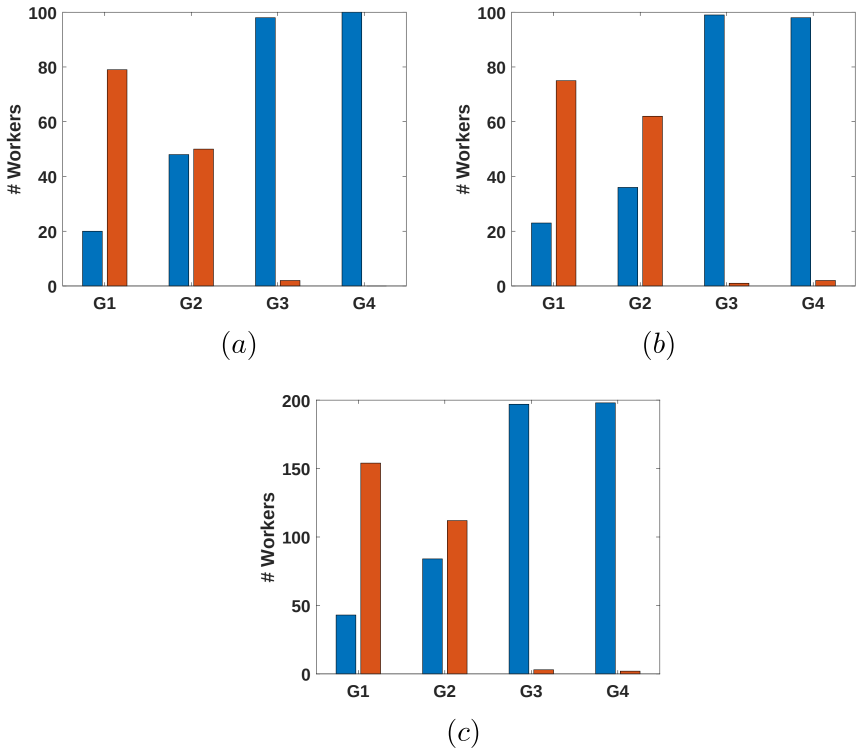

Figure 9: **Number of workers' correct and incorrect replies in all four subtasks for HIT majority. (a) Results collected from Study 1, (b) results collected from Study 2, (c) aggregated results collected** We present with the blue color (left) bar the correct replies and with the red color (right) bar the incorrect replies.

Table 4: **The correlation coefficient of the worker's correct response ratio (in the four subtasks) with the response time in each graph, for Study 1.** Columns represent the correlation coefficient for each of the graph and rows represent the HIT task.

	G1	G2	G3	G4
Color	0.2852	0.1945	0.0478	-0.0013
Majority	0.4905	0.3791	0.1270	0.2196
Count	0.4083	0.3009	0.4040	0.0969

Table 5: **The correlation coefficient of the worker's correct response ratio (in the four subtasks) with the response time in each graph, for Study 2.** Columns represent the correlation coefficient for each of the graph and rows represent the HIT task.

	G1	G2	G3	G4
Color	0.4044	0.4419	0.4046	0.2784
Majority	0.4133	0.3239	0.1124	0.1239
Count	0.4765	0.3656	0.2539	0.1738

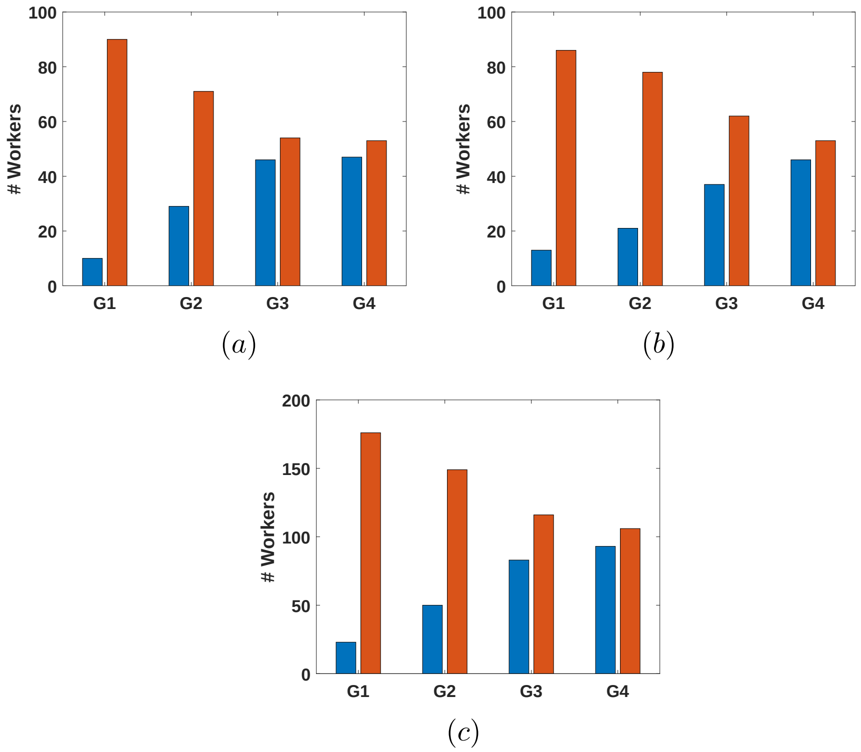

Figure 10: **Number of workers' correct and incorrect replies in all four subtasks for HIT count. (a) Results collected from Study 1, (b) results collected from Study 2, (c) aggregated results collected** We present with the blue color (left) bar the correct replies and with the red color (right) bar the incorrect replies. In all four graphs the correct answer is equal to the mode.

Table 6: **The correlation coefficient of the worker's correct response ratio (in the four subtasks) with the response time in each graph, for the aggregate of the two studies.** Columns represent the correlation coefficient for each of the graph and rows represent the HIT task.

	G1	G2	G3	G4
Color	0.3216	0.3345	0.2563	0.1530
Majority	0.4438	0.3347	0.1256	0.1362
Count	0.4446	0.3365	0.3284	0.1423

Table 7: **The correlation coefficient of the worker's correct response ratio (in the four subtasks) with the absolute distance to the correct value for HIT count.** Columns represent the correlation coefficient for each of the graph. The first two rows represent the results for the first and second round of experiments and the third row the results when aggregating the collected data of the two rounds.

Experiment Round	G1	G2	G3	G4
Study 1	-0.1591	-0.3359	-0.2559	-0.2094
Study 2	-0.3689	-0.2824	-0.1627	-0.1547
Aggregate	-0.1791	-0.3136	-0.1858	-0.1805

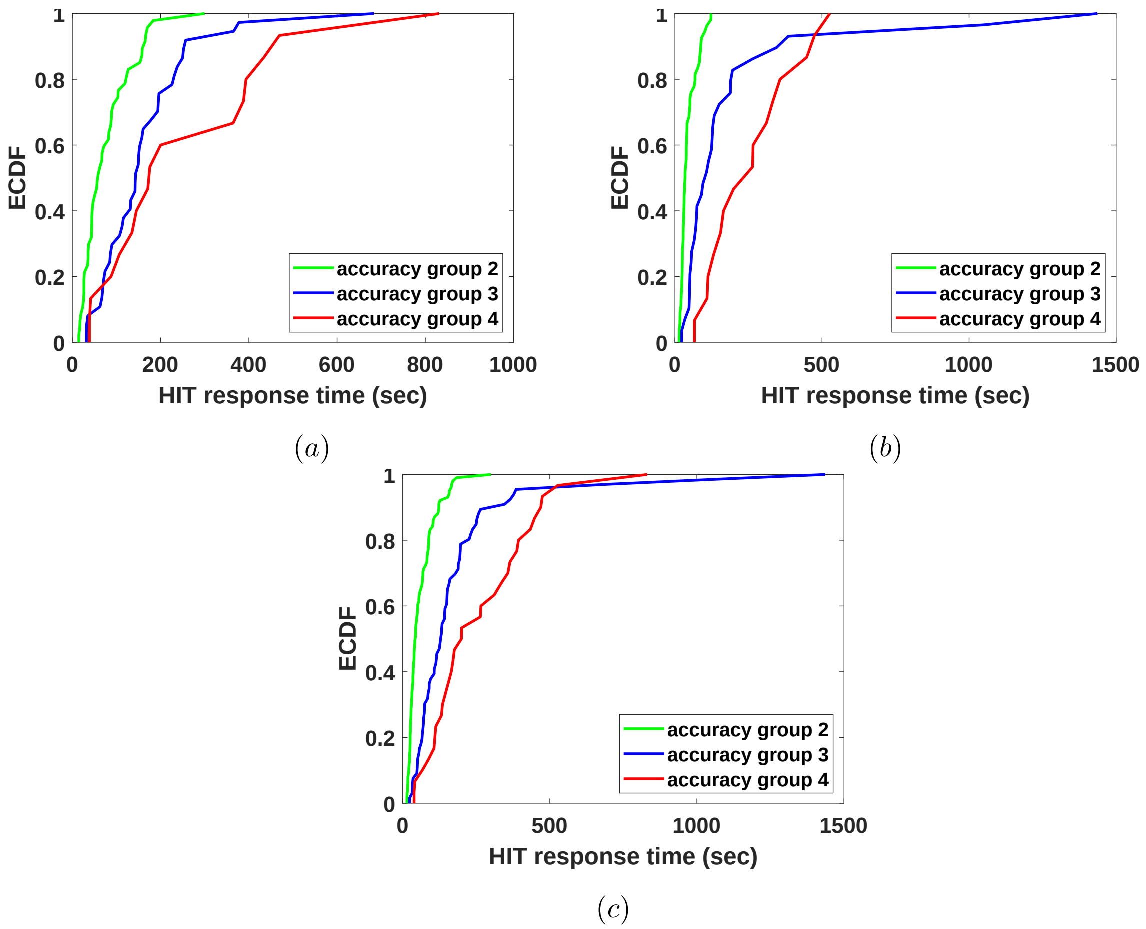

Figure 11: **The ECDF of the total response time for HIT majority. (a) Results collected from Study 1, (b) results collected from Study 2, (c) aggregated results collected.** From left to right, the workers with accuracy degree two up to degree four.

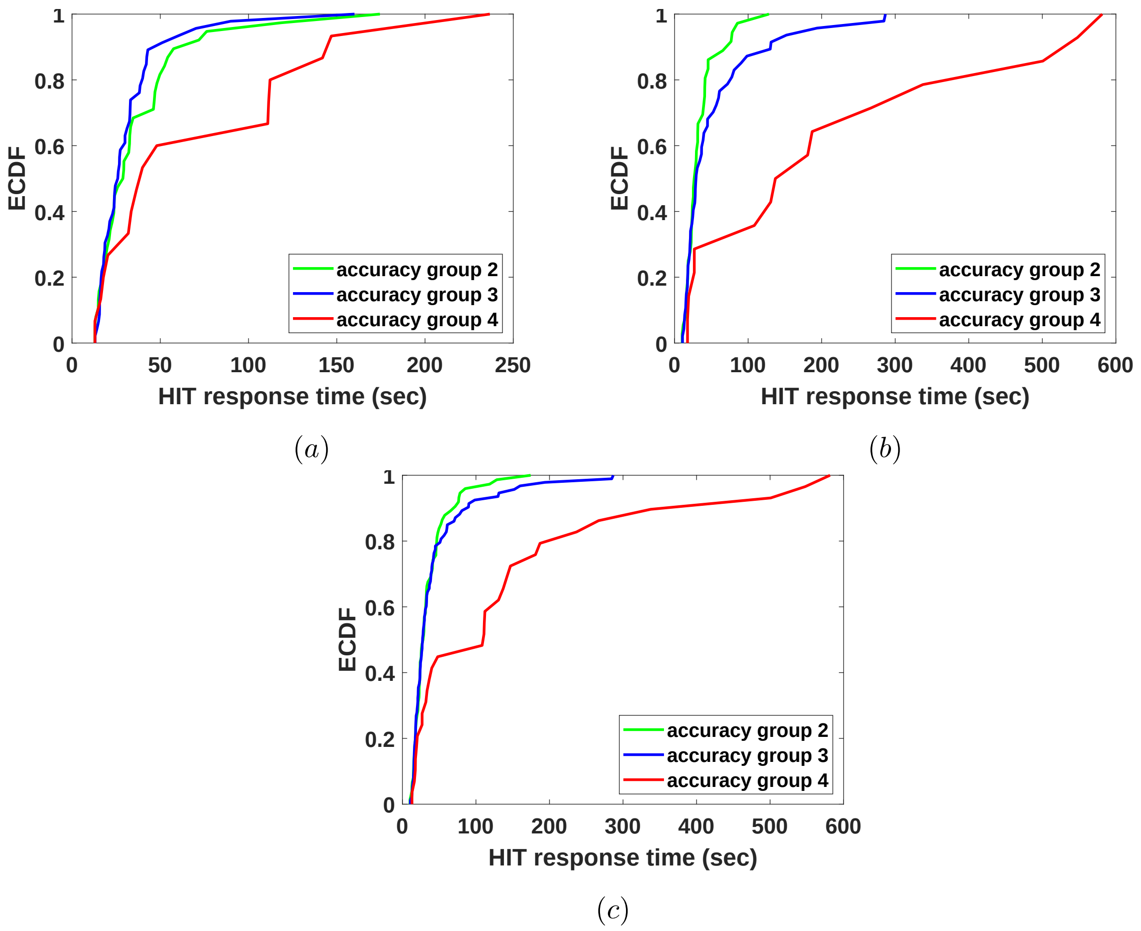

Figure 12: **The ECDF of the total response time for HIT color for the three accuracy groups. (a) Results collected from Study 1, (b) results collected from Study 2, (c) aggregated results collected.**

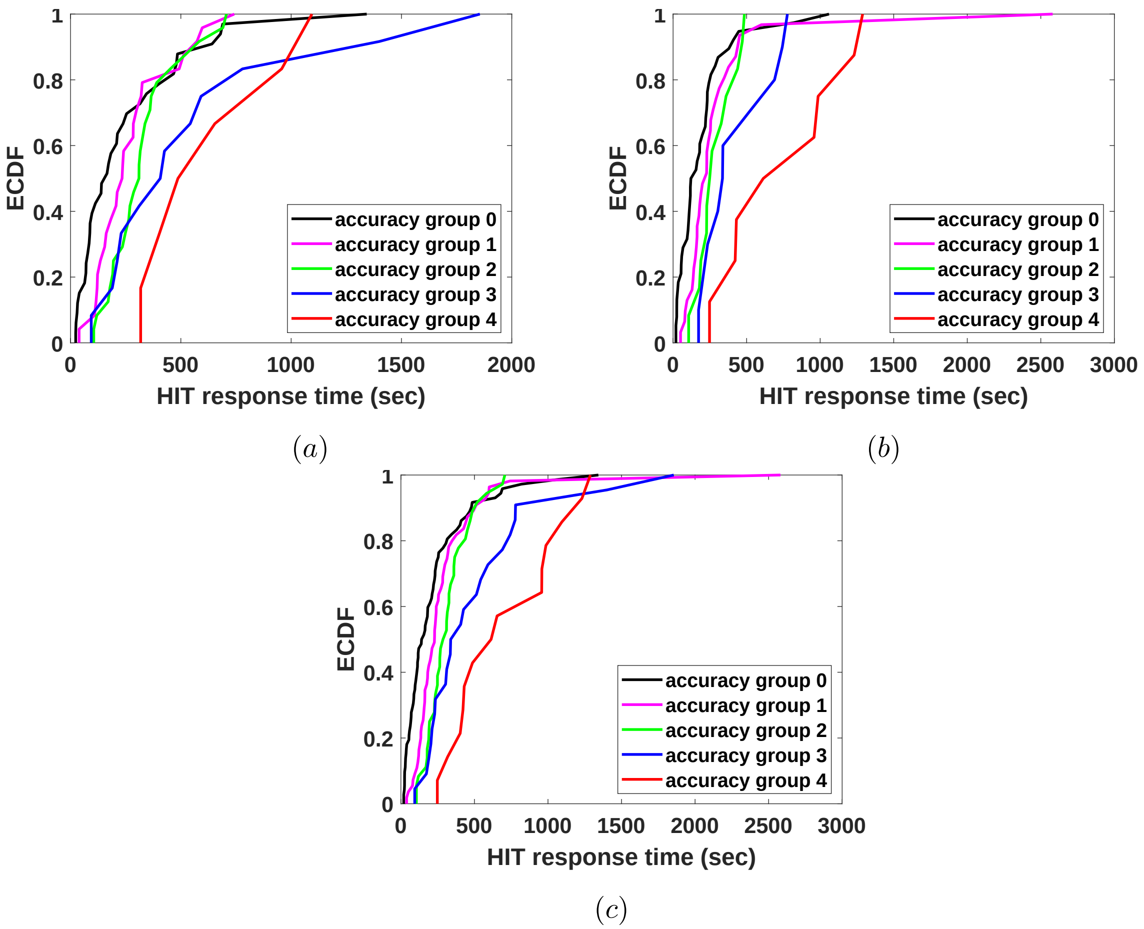

Figure 13: **The ECDF of the total response time for HIT count for the five accuracy groups. (a) Results collected from Study 1, (b) results collected from Study 2, (c) aggregated results collected.**

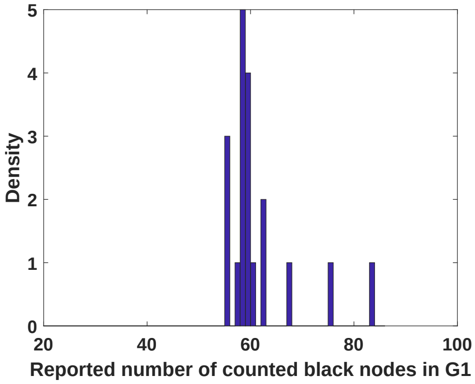

(a)

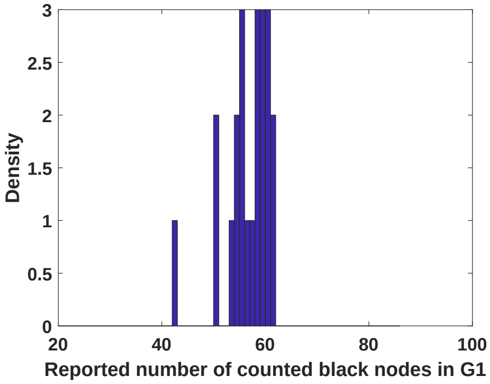

(b)

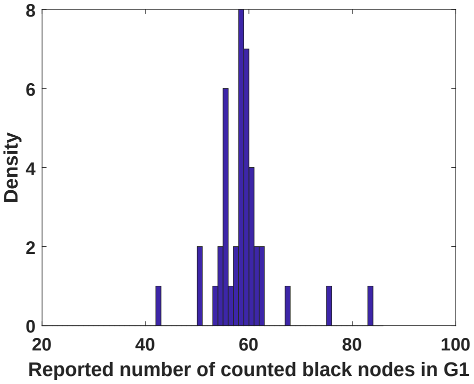

(c)

Figure 14: **Histogram of the density of reported number of black nodes in graph G1 for HIT majority. (a) Results collected from Study 1, (b) results collected from Study 2, (c) aggregated results collected.**

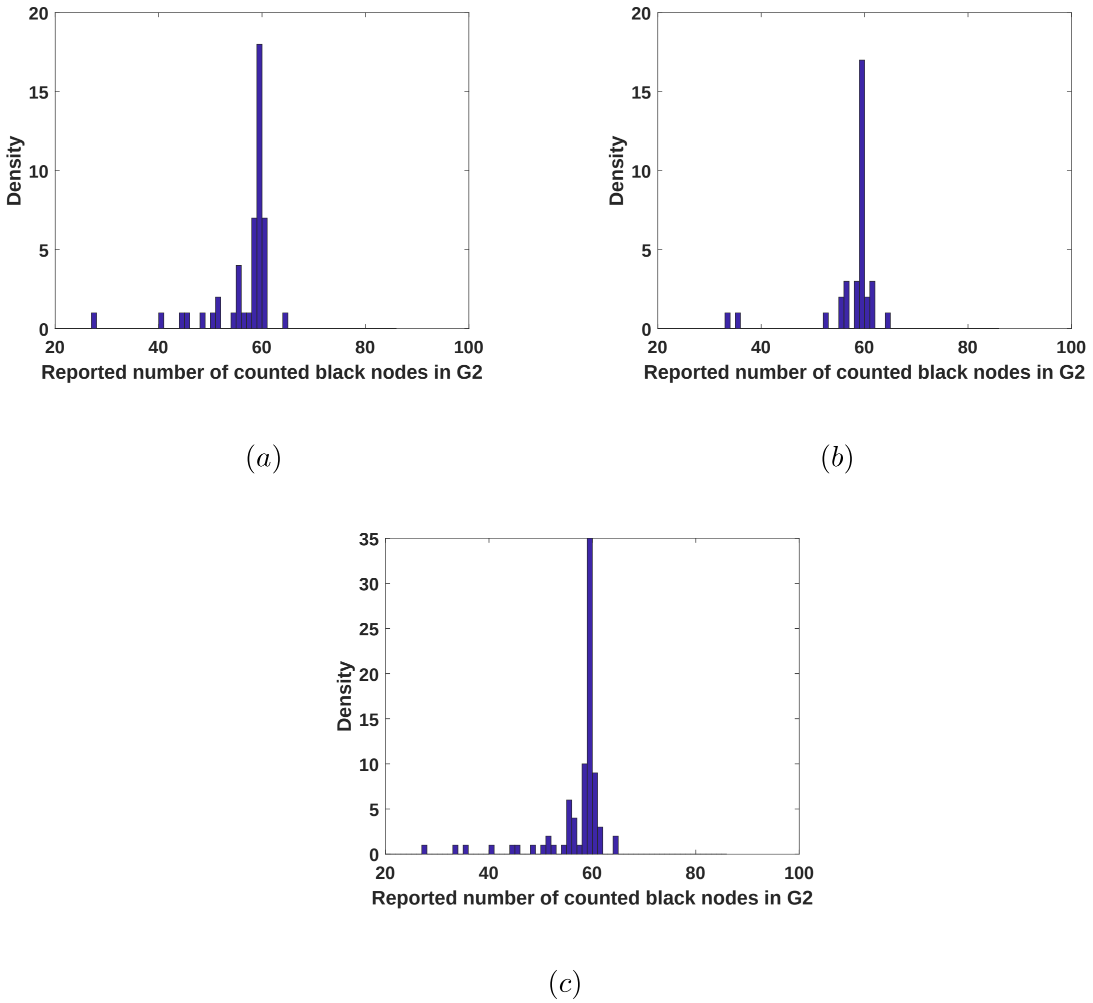

Figure 15: **Histogram of the density of reported number of black nodes in graph G2 for HIT majority. (a) Results collected from Study 1, (b) results collected from Study 2, (c) aggregated results collected.**

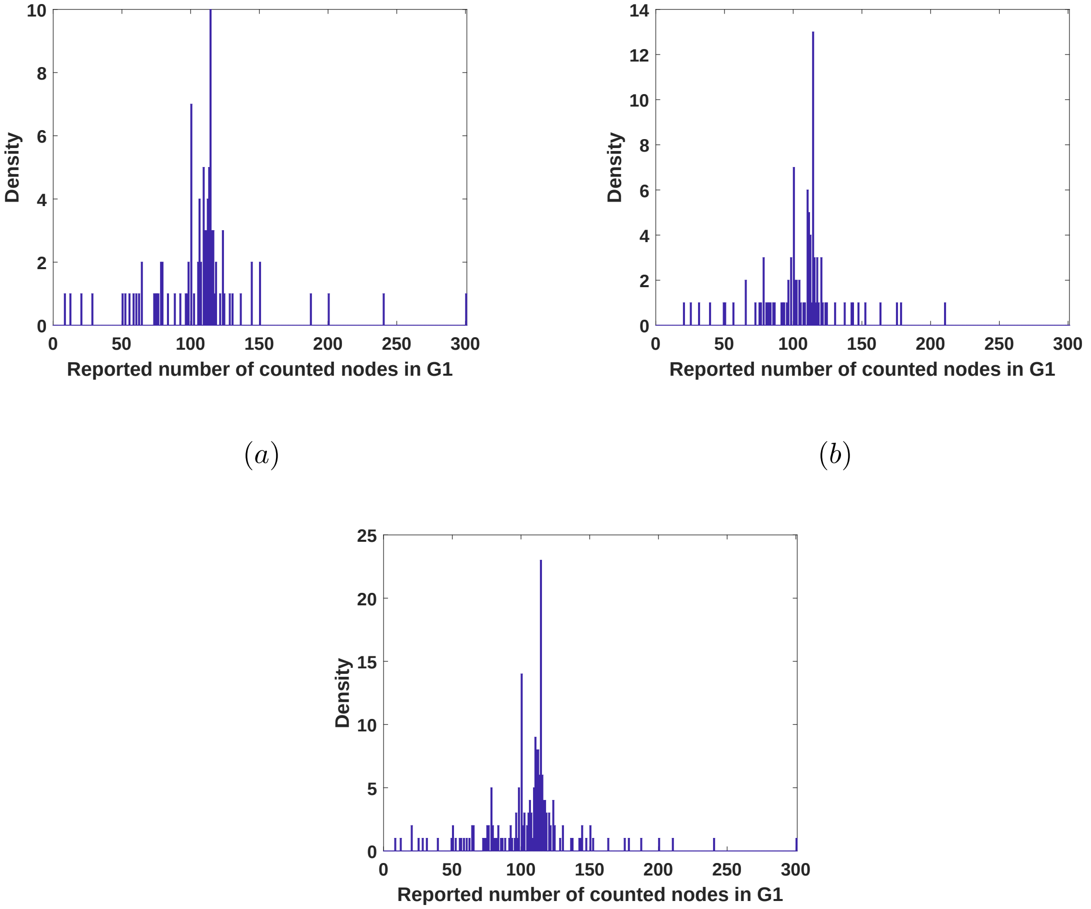

(a) (b)

(c)

Figure 16: **Histogram of the density of reported number of nodes in graph G1 for HIT count.** The correct answer is 114.

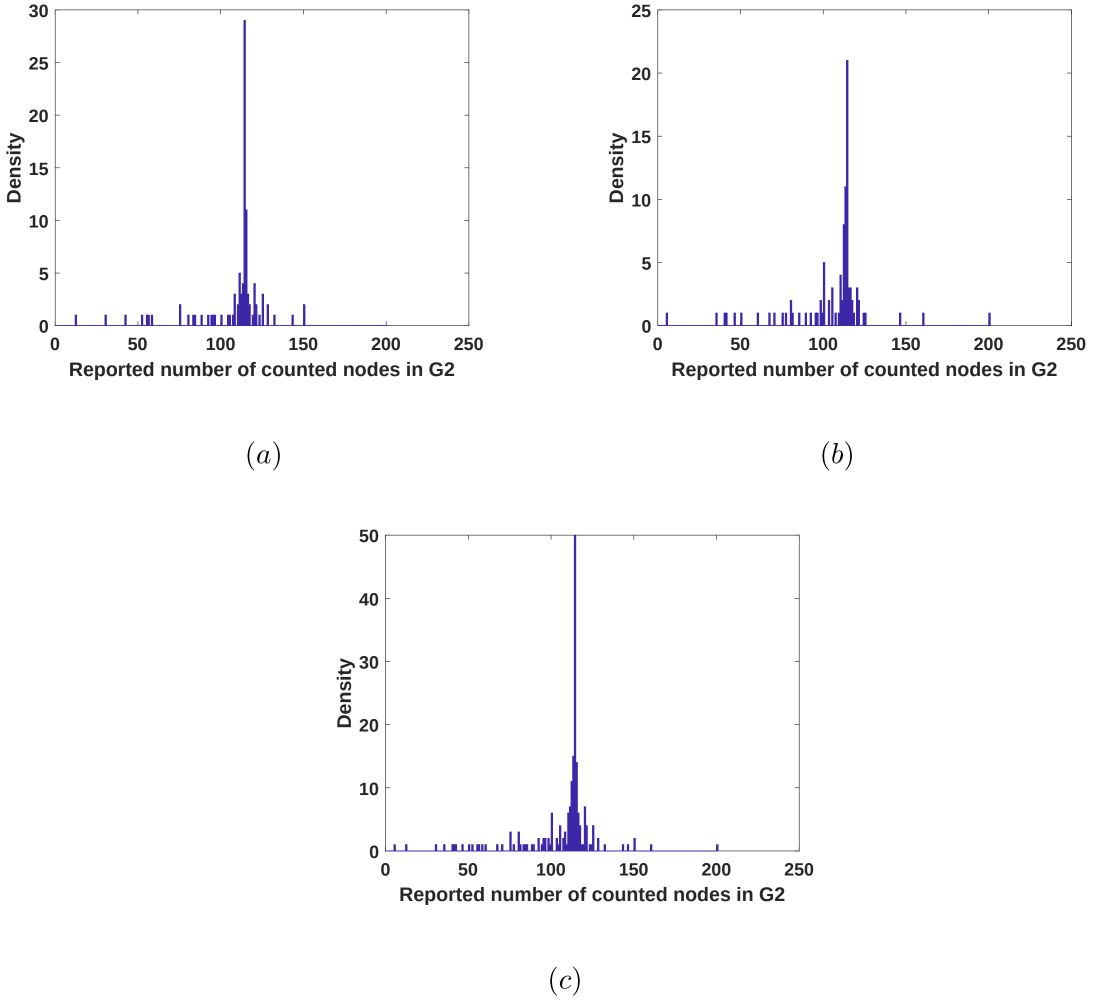

Figure 17: **Histogram of the density of reported number of nodes in graph G2 for HIT count.**The correct answer is 114.

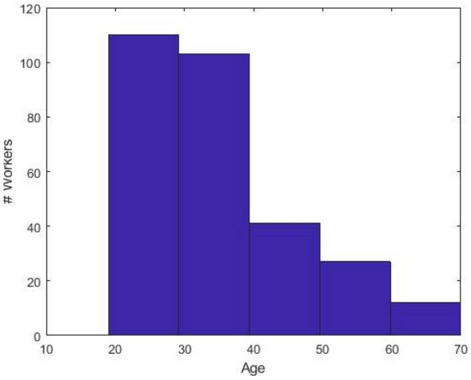

(a)

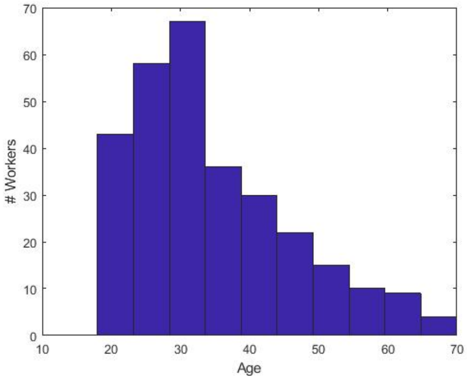

(b)

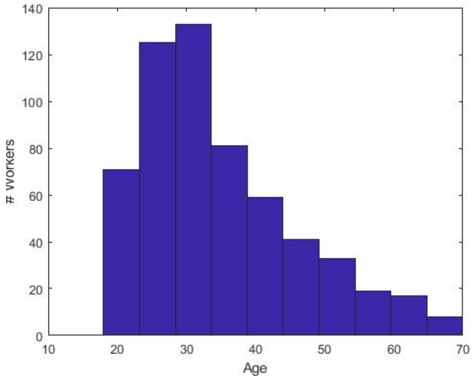

(c)

Figure 18: **The reported age of participating workers in all three HITs. (a) data collected from Study 1, (b) data collected from Study 2, (c) data collected from both rounds of experiments**

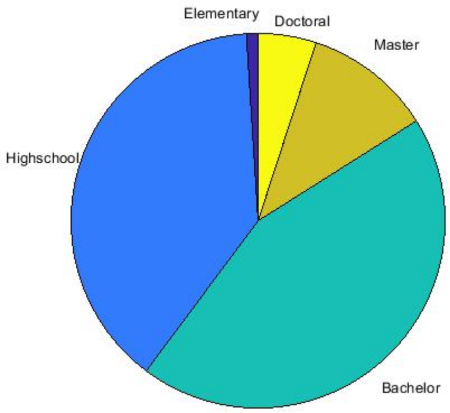

(a)

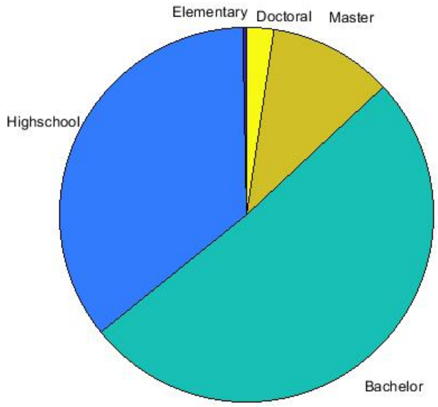

(b)

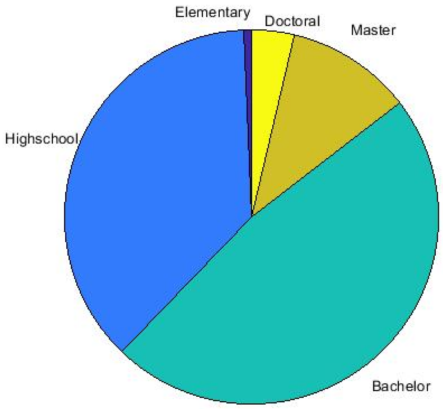

(c)

Figure 19: **The reported education of participating workers in all three HITs. (a) data collected from Study 1, (b) data collected from Study 2, (c) data collected from both rounds of experiments**

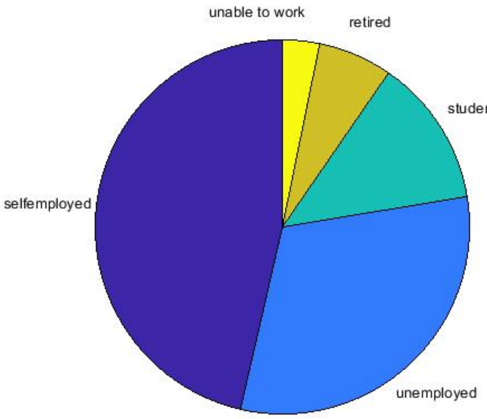

(a)

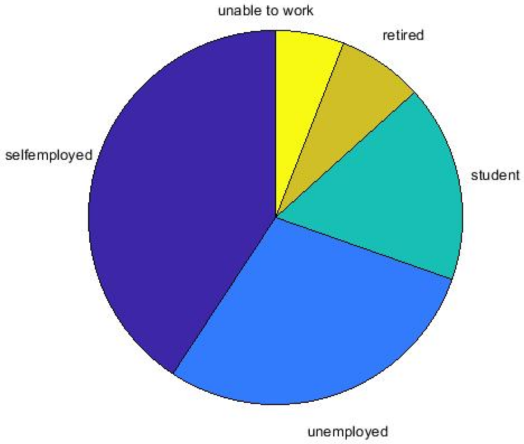

(b)

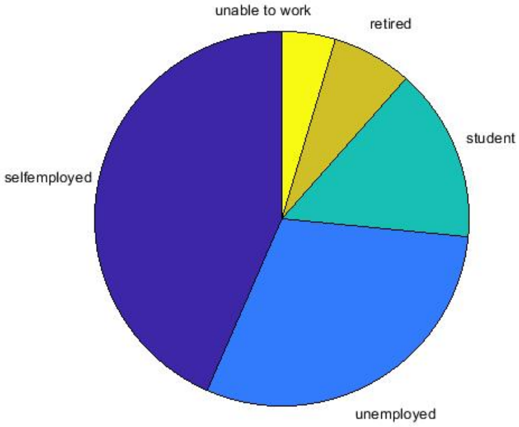

(c)

Figure 20: **The reported occupation of participating workers in all three HITs. (a) data collected from Study 1, (b) data collected from Study 2, (c) data collected from both rounds of experiments**

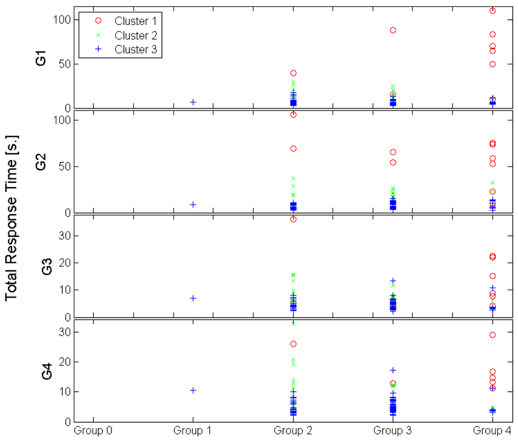

(a)

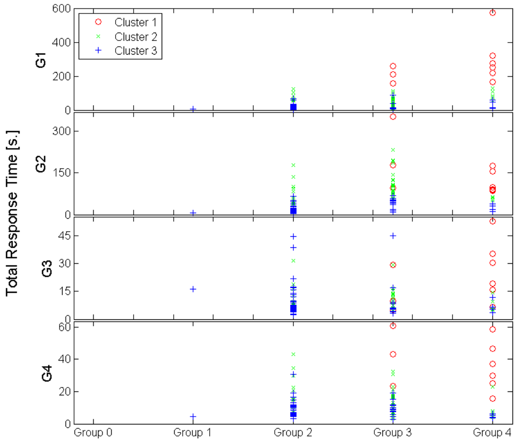

(b)

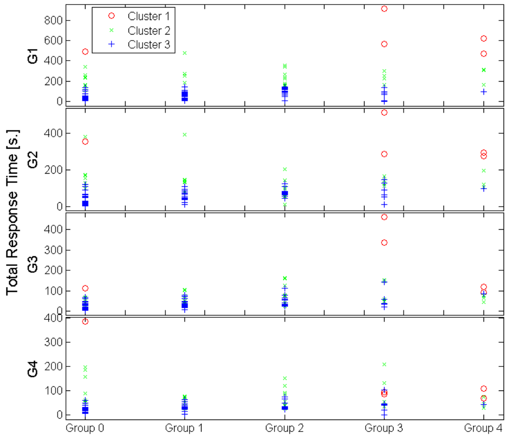

(c)

Figure 21: **Scatter plot of the workers' response time against workers accuracy group for (a) the four HIT color subtasks, (b) the four HIT majority subtasks, and (c) the four HIT count subtasks.**
